# Injectable Hydrogels for the Treatment of Temporomandibular Joint Osteoarthritis: From Tissue-Engineering Scaffolds to Joint Lubricants and Mechanical Buffers

**DOI:** 10.3390/pharmaceutics18080949

**Published:** 2026-07-31

**Authors:** Chen Huang, Yang Yuan, Zhuofan Yu, Xu Feng, Bowen Zheng, Yi Liu

**Affiliations:** Department of Orthodontics, Clinical Medical Research Center of Orthodontic Disease, School and Hospital of Stomatology, China Medical University, No. 117 Nanjing North Street, Heping District, Shenyang 110002, Chinayyang2025@cmu.edu.cn (Y.Y.); liuyi@cmu.edu.cn (Y.L.)

**Keywords:** temporomandibular joint osteoarthritis, injectable hydrogels, intra-articular delivery, tissue engineering, mechanical buffering

## Abstract

**Introduction/Objectives:** Temporomandibular joint osteoarthritis (TMJOA) causes pain, mandibular dysfunction, fibrocartilage degradation, and synovial inflammation. Current therapies are mainly palliative and limited by rapid intra-articular clearance and insufficient disease-modifying effects. This review summarizes injectable hydrogels for TMJOA as regenerative scaffolds, drug delivery systems, joint lubricants, and mechanical buffers. **Methods:** Relevant studies were identified from PubMed/MEDLINE, Web of Science and Google Scholar using terms related to TMJOA, injectable hydrogels, intra-articular delivery, tissue engineering, cartilage repair, lubrication, viscosupplementation and mechanical buffering. Studies were selected if they addressed hydrogel design, biological function, mechanical performance, biosafety or translational evaluation. **Results:** Injectable hydrogels have been investigated mainly as bioactive regenerative scaffolds and acellular functional biomaterials. Bioactive systems may regulate inflammation and oxidative stress, deliver cells, exosomes, drugs or growth factors, and support fibrocartilage repair. Acellular systems primarily aim to improve intra-articular retention, lubrication, viscoelastic adaptation and mechanical buffering. However, current evidence remains largely preclinical, with limited validation of long-term residence, degradation behavior, TMJ-specific mechanical performance, repeat dosing, biosafety and functional outcomes. **Conclusions:** Injectable hydrogels represent promising multifunctional platforms for TMJOA treatment. Cell-, exosome-, and growth factor-loaded systems show regenerative potential but face manufacturing, safety, regulatory, and long-term validation challenges. Acellular multifunctional hydrogels may be more feasible for near-term translation. **Clinical Significance**: Injectable hydrogels may provide minimally invasive, locally sustained treatment for TMJOA by integrating symptom control, microenvironment modulation, and mechanical adaptation.

## 1. Introduction

Temporomandibular joint osteoarthritis (TMJOA) is a clinically important subtype of temporomandibular disorders (TMD) and a major cause of pain, joint dysfunction, and reduced oral health-related quality of life. Globally, it is estimated to affect approximately 8–16% of the general population [1,2,3]. It is a chronic degenerative joint disease centered on fibrocartilage deterioration of the mandibular condyle and is accompanied by sterile inflammation involving multiple joint-associated tissues, including the synovium, joint capsule, and periarticular muscles. At the pathological level, TMJOA is characterized by chondrocyte death and progressive extracellular matrix (ECM) breakdown, together with a persistent catabolic cascade driven by matrix-degrading enzyme systems, particularly matrix metalloproteinases (MMPs) and a disintegrin and metalloproteinase with thrombospondin motifs (ADAMTS). Clinically, patients often present with pain, joint sounds, swelling, and limited mouth opening, highlighting TMJOA as not only a structural joint disease but also a functionally relevant condition in oral medicine [2,3].

Current management of TMJOA, especially in the early stages, is still dominated by conservative interventions. These include pharmacological approaches, such as nonsteroidal anti-inflammatory drugs (NSAIDs), disease-modifying osteoarthritis drugs (DMOADs), glucocorticoids, and biologics, as well as non-pharmacological modalities, including naturopathy, thermotherapy, and electromagnetic therapy [4,5]. However, most of these interventions remain primarily symptomatic rather than disease-modifying, and effective strategies capable of reliably halting structural degeneration and progressive functional impairment while maintaining acceptable tolerability are still lacking [6]. Moreover, after intra-articular administration, many small-molecule agents are rapidly cleared through synovial capillaries and lymphatic drainage, making it difficult to sustain therapeutically effective local concentrations. This pharmacokinetic limitation not only restricts treatment efficacy but also reduces patient compliance. Although surgical reconstruction may be considered in advanced cases, the complex pathogenesis of TMJOA and the unfavorable local microenvironment often limit the long-term success of single-modality interventions in achieving durable structural repair and functional recovery [7].

Recent advances in tissue engineering and biomaterials have created new opportunities for TMJOA treatment. In the broader field of oral and maxillofacial regeneration, multifunctional nanomaterials such as halloysite nanotubes and functionalized nanodiamonds have been explored as drug carriers, bioactive interfaces, and scaffold components for personalized therapeutic applications [8,9]. Among the emerging biomaterial platforms, hydrogels have attracted considerable attention owing to their highly hydrated three-dimensional network structure, favorable biocompatibility, biodegradability, and capacity to serve as carriers for cells and bioactive cargos. In particular, injectable hydrogels can be delivered in a minimally invasive manner and undergo in situ gelation, making them especially attractive for cartilage repair and intra-articular applications [10]. Nevertheless, injectability alone does not guarantee prolonged therapeutic efficacy within the joint space. In the temporomandibular joint, which is characterized by a small articular cavity and frequent cyclic rotational and translational motion during daily functions such as mastication, speaking, and swallowing, biomaterials are particularly vulnerable to synovial fluid dilution, leakage or migration, and premature degradation. These challenges are further amplified by the unique anatomical architecture, histological characteristics, and complex biomechanical environment of the temporomandibular joint, thereby placing higher demands on the rational design of injectable hydrogel systems for TMJOA [7].

**Figure 1:**
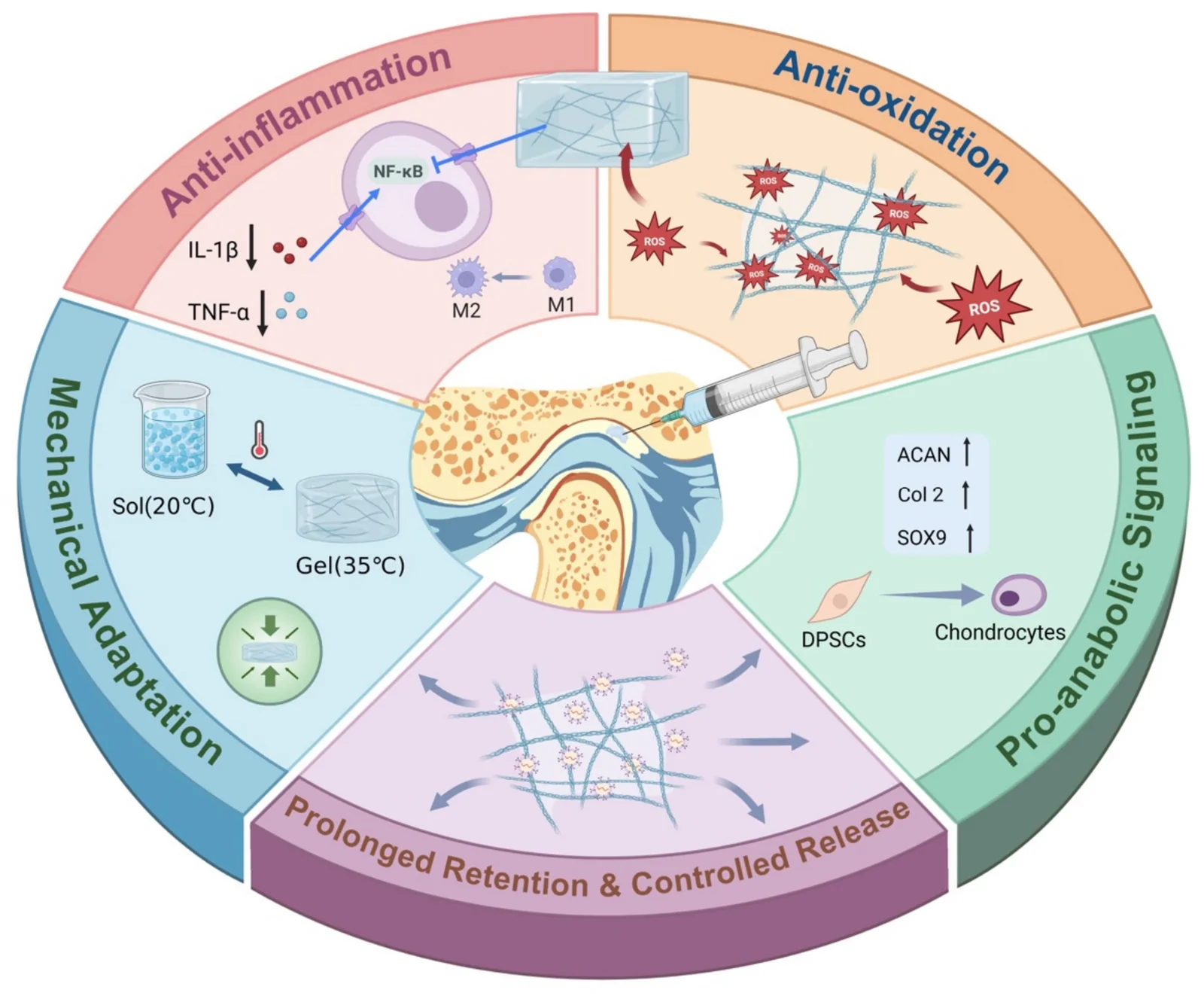
Functional landscape of injectable hydrogels for temporomandibular joint osteoarthritis (TMJOA). The schematic summarizes the major therapeutic roles of injectable hydrogels in TMJOA, including microenvironment modulation, controlled delivery, cartilage repair, intra-articular retention, and mechanical adaptation. Abbreviations: reactive oxygen species (ROS); dental pulp stem cells (DPSCs).

Against this background, injectable hydrogels are being increasingly explored to address two major clinical needs in TMJOA. First, as injectable tissue-engineering scaffolds, they can be engineered to deliver cells, exosomes, growth factors, or therapeutic agents, thereby modulating the local inflammatory and oxidative stress microenvironment, suppressing matrix degradation, and promoting cartilage regeneration [11]. Second, by virtue of their lubrication performance and mechanical buffering capacity, injectable hydrogels may reduce frictional wear during joint movement and improve load adaptation through the optimization of viscoelasticity and intra-articular retention [10]. Accordingly, this review summarizes recent progress in the design of injectable hydrogel systems for TMJOA from the perspective of material synthesis and functional integration. Using a framework centered on functional role, key material properties, and experimental validation, we further discuss the design logic, evaluation endpoints, and translational potential of different hydrogel platforms, with the aim of providing insight into material selection and combinatorial therapeutic strategies for TMJOA. As summarized in Figure 1, the current therapeutic logic of injectable hydrogels for TMJOA extends beyond simple injectability and increasingly emphasizes the integration of microenvironment modulation, controlled delivery, and mechanical adaptation.

Rather than providing an exhaustive catalogue of individual hydrogel formulations, this review organizes current evidence according to the translational functions of injectable hydrogels in TMJOA, including microenvironment modulation, bioactive cargo delivery, tissue-regenerative scaffolding, lubrication, mechanical buffering, and intra-articular retention.

## 2. Unique Design Constraints and Therapeutic Rationales for Injectable Hydrogels in TMJOA

The application of injectable hydrogels to treat temporomandibular joint osteoarthritis (TMJOA) cannot simply extrapolate the paradigms established for large, weight-bearing joints like the knee. A successful strategy hinges on a profound understanding of the unique design constraints imposed by the TMJ, which stem from its distinct anatomical, biomechanical, and microenvironmental characteristics.

Anatomically, the TMJ cavity has a volume of only a few milliliters and is divided by the articular disc into separate superior and inferior compartments [12]. This not only limits the injectable volume but also complicates precise delivery. Furthermore, its articular surfaces are covered by fibrocartilage, not hyaline cartilage, which imposes distinct requirements for material biocompatibility and regenerative strategies [13]. Biomechanically, the TMJ is characterized by frequent, repetitive rotational and translational movements associated with daily oral functions [14]. This environment continuously exposes injected materials to dynamic shear stresses, making them highly susceptible to mechanical washout and migration. From a microenvironmental perspective, the rapid turnover of synovial fluid leads to a much faster clearance rate for small-molecule drugs and uncrosslinked materials compared to larger joints, posing a formidable challenge to the in situ residence stability of hydrogels and the durability of their therapeutic payload release [15].

**Figure 2:**
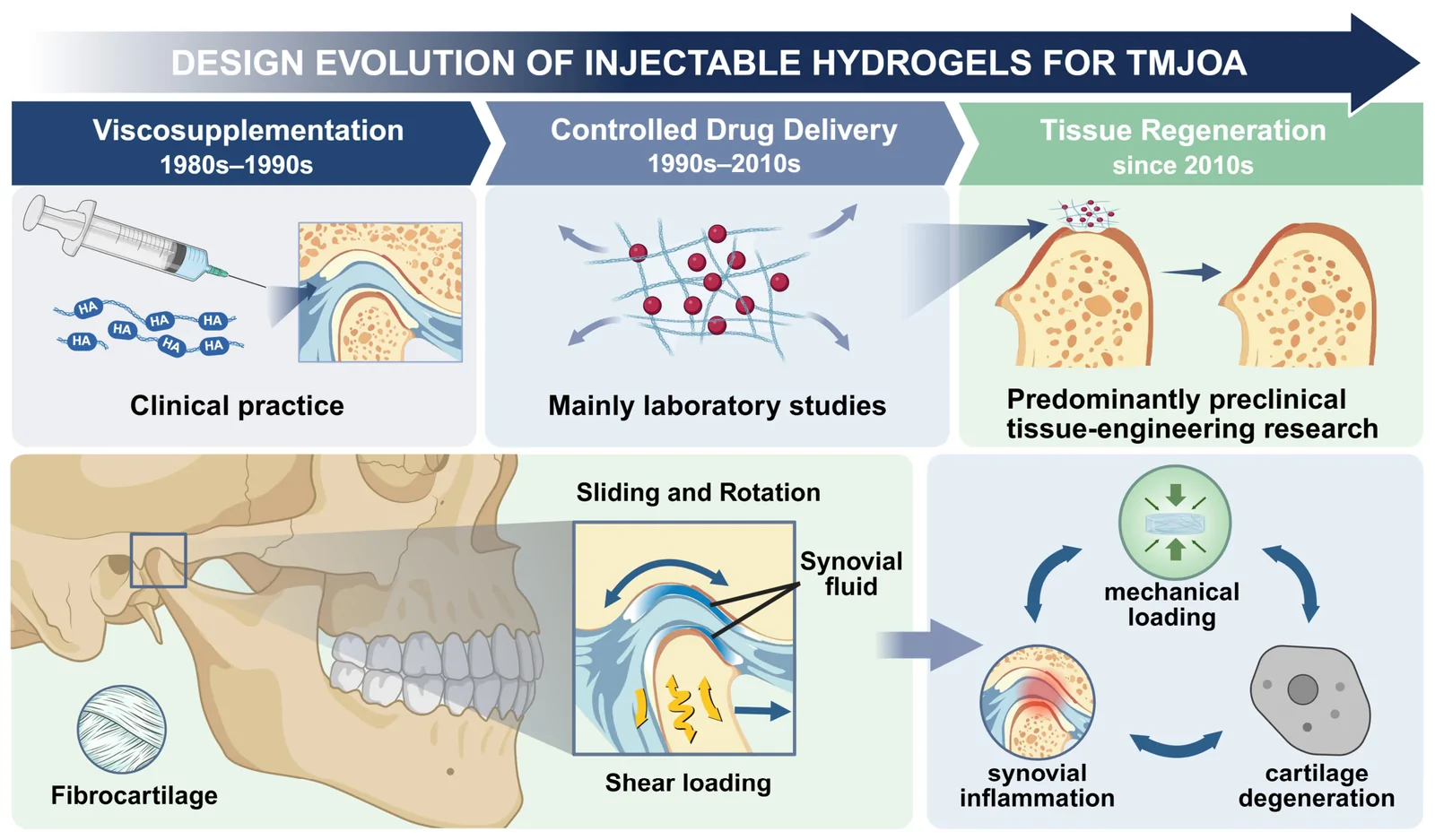
TMJ-specific constraints and design evolution of injectable hydrogels for TMJOA. The schematic outlines the progression from viscosupplementation to controlled delivery and regenerative hydrogel strategies, together with key TMJ-specific constraints, including fibrocartilaginous surfaces, limited cavity volume, rapid synovial turnover, and repetitive rotational–translational motion.

Collectively, these factors shape the tightly coupled pathological triad of TMJOA: abnormal mechanical loading, synovial inflammation, and cartilage degeneration [16]. It is precisely this understanding that has driven the logical evolution of injectable therapies for TMJOA, transitioning from macroscopic functional replacement to microenvironmental intervention. Early strategies, such as viscosupplementation with hyaluronic acid, focused on restoring the lubricating and buffering functions of synovial fluid for short-term symptomatic relief [17]. However, the rapid clearance of conventional drugs like corticosteroids limited their therapeutic longevity [18], which spurred the development of hydrogel-based systems for sustained drug delivery, aiming to prolong the effective residence time of therapeutic agents within the joint cavity [19]. More recently, advances in tissue engineering have redefined the role of hydrogels, shifting their function from passive carriers to active regenerative scaffolds tasked with modulating cell behavior and promoting tissue repair [7]. This progression has culminated in the current landscape, which is defined by three principal design rationales: joint lubrication/mechanical buffering, sustained drug delivery, and tissue regeneration. As illustrated in Figure 2, the design evolution of injectable hydrogels for TMJOA is closely linked to the unique anatomical, biomechanical, and microenvironmental constraints of the temporomandibular joint.

## 3. Engineering Design Strategies, Key Performance Metrics, and Evaluation Frameworks for Injectable Hydrogels

Under the unique constraints of TMJOA, the central challenge for engineering injectable hydrogels lies in maintaining their structural integrity and functional longevity against the combined challenges of synovial fluid dilution, repetitive motion-induced shear, and physiological clearance. This necessitates a systematic balance between clinical operability (e.g., shear-thinning behavior and post-injection self-healing) and a suite of key performance metrics. These metrics constitute the engineering foundation for assessing the suitability of a hydrogel system for TMJOA and primarily include the following: (1) In situ gelation kinetics: the ability to form a stable gel rapidly and reproducibly within the synovial environment [20]. (2) In vivo residence stability: demonstrating excellent resistance to dilution and washout to guarantee a sufficient therapeutic duration [21]. (3) Mechanical durability under cyclic loading: maintaining viscoelastic stability and fatigue resistance under repetitive shear associated with TMJ motion. (4) Tribological performance: the capacity to form a stable lubricating layer at the articular interface to reduce friction and wear [22]. (5) Biocompatibility: ensuring that both the material and its degradation products are well-tolerated by host tissues [23]. (6) Congruent release kinetics: matching the release profile of bioactive agents with the temporal window required for pathological modulation or tissue repair [24].

Guided by these performance targets, a variety of function-oriented engineering strategies have been developed. These include introducing dynamic reversible bonds or dual-network structures to enhance mechanical toughness and fatigue resistance [2,5]; designing microsphere/microgel composite systems to improve lubrication while enabling sustained drug release [25]; and constructing smart networks responsive to the pathological microenvironment (e.g., pH, enzymes) for targeted and on-demand delivery [26]. However, validating the efficacy of these designs necessitates a comprehensive evaluation framework that integrates rheological, mechanical, tribological, in vivo behavioral, and therapeutic outcome assessments. Among these, the two performance indicators most critical for predicting clinical translation are the in vivo residence time, directly verified by techniques such as live imaging, and the mechanical and tribological stability under conditions that faithfully simulate the frequent cyclic rotational and translational motions of the TMJ during daily oral functions.

A critical assessment of the current literature reveals that a central bottleneck in this field lies not only in the continued introduction of new material formulations, but also in the lack of standardized evaluation systems tailored to the specific mechanical and biological environment of the TMJ [27]. The majority of current performance data is derived from simplified in vitro setups or large-joint animal models, which fail to adequately replicate the extreme “small-cavity, high-shear” conditions of the TMJ. Consequently, the current evidence highlights a methodological gap between material development and clinically relevant evaluation. Most current performance data are derived from simplified in vitro setups or large-joint animal models, which do not adequately replicate the small-cavity, high-shear conditions of the TMJ. This limitation complicates direct comparison among different hydrogel systems and restricts accurate assessment of their in situ residence, structural integrity under repetitive motion-induced shear, and therapeutic efficacy for condylar fibrocartilage repair. Therefore, rather than providing an exhaustive catalogue of individual hydrogel formulations, this review organizes current evidence according to the translational functions of injectable hydrogels in TMJOA, including microenvironment modulation, bioactive cargo delivery, tissue-regenerative scaffolding, lubrication, mechanical buffering, and intra-articular retention.

## 4. Polymeric Platforms for Injectable Hydrogels in TMJOA: Material Types, Properties, and Selection

The therapeutic behavior of an injectable hydrogel is ultimately governed by its constituent polymer, which dictates gelation, mechanical strength, degradation rate, bioactivity, and compatibility with therapeutic cargo. Reflecting this, the hydrogels developed for TMJOA are constructed from a limited set of polymer families that are conventionally divided into naturally derived and synthetic backbones, frequently combined within composite networks [7]. As a general rule, natural polymers offer excellent biocompatibility and biodegradability but limited mechanical strength, whereas synthetic polymers provide tunable and reproducible mechanical and degradation profiles at the expense of intrinsic bioactivity [7]. A clear understanding of the origin, advantages, and limitations of each material is therefore a prerequisite for the rational selection and combination of polymers for TMJOA.

Among naturally derived polymers, hyaluronic acid (HA) is the most established. As a native component of synovial fluid and the cartilage extracellular matrix, it is biocompatible and intrinsically lubricating, and it underpins both conventional viscosupplementation (see Section 6) and many engineered TMJOA hydrogels [1,17,26,28,29,30,31,32,33,34]. Its principal drawbacks, namely rapid enzymatic clearance and weak mechanical properties, motivate chemical modification and crosslinking [7]. Gelatin and its photocrosslinkable derivative gelatin methacryloyl (GelMA), both derived from collagen, retain cell-adhesive motifs and offer tunable stiffness and have served as microsphere and scaffold platforms for cell- and drug-loaded TMJOA repair [1,28]. Chitosan, a cationic polysaccharide, contributes mucoadhesive, antibacterial, and thermosensitive behavior [28] and has featured among the injectable drug-delivery systems evaluated in TMJOA models [35]. Silk fibroin, valued for its strong and tunable mechanics and low immunogenicity, can be rendered photocrosslinkable as methacrylated silk fibroin (SilMA) to deliver chondrogenic agents for cartilage regeneration [32]. Alginate, an ionically gelling polysaccharide, is non-immunogenic and mild to process but shows limited cell adhesion and less controllable degradation, and has been engineered into stimulus-responsive networks for osteoarthritis [26].

Synthetic polymers are selected when defined, reproducible, and tunable properties are required. Poly(ethylene glycol) (PEG) and aliphatic polyesters such as (lactic-co-glycolic acid) PLGA and (L-lactide-co-ε-caprolactone) PLCL provide adjustable mechanical strength and degradation kinetics and have been incorporated into composite injectable systems for TMJ condylar repair, for example, a 3B-PEG/Nell-1 construct [31]; the degradation rate of such polyesters can be tuned over a wide range by adjusting their composition [7]. Poloxamers (e.g., Pluronic F127) undergo a thermosensitive sol–gel transition that enables in situ gelation upon injection, but they form mechanically weak, rapidly eroding, and poorly biodegradable gels, which limits their standalone use [36]. Because no single polymer simultaneously satisfies the demands of injectability, intra-articular retention, mechanical durability, and bioactivity in the TMJ (Section 3), most contemporary designs are composites that pair a bioactive natural component with a mechanically or responsively engineered synthetic one, as illustrated by tilapia gelatin/HA [1] and dual-responsive GelMA/F127DA [37] systems. The principal polymers, their properties, and representative TMJOA-relevant applications are summarized in Table 1.

## 5. Functionalized Hydrogels for Tissue Engineering: From Passive Support to Active Reprogramming

In the context of regenerative medicine for TMJOA, the role of injectable hydrogels is evolving from that of a passive void-filler to an engineered platform capable of actively integrating multiple biological functions to reprogram the pathological microenvironment. More broadly, advances in scaffold fabrication, including three-dimensional printing of fiber-based natural biomaterials, have expanded the design space for tissue-engineering matrices with tunable architecture, mechanical properties, and biological functionality [47]. This paradigm shift is driven by the profound recognition that mono-functional materials are ill-equipped to address the confluence of challenges in the TMJ: repetitive motion-induced mechanical stress, rapid synovial fluid clearance, and persistent inflammatory and oxidative insults. Consequently, modern hydrogel design is converging on multifunctional synergy, aiming to bridge the gap from symptomatic control to structural repair through a combination of strategies, including immune modulation, antioxidant defense, delivery of pro-anabolic signals, sustained-release potentiation, and biomechanical matching [48]. Accordingly, this section discusses injectable hydrogels according to their major functional roles, including immunomodulatory platforms, responsive therapeutic systems, carriers for drugs and bioactive factors, cell-laden tissue-engineering scaffolds, and matrices for three-dimensional culture and disease modeling.

### 5.1. Hydrogels as Immunomodulatory and Anti-Inflammatory Platforms

Given that the inflammatory microenvironment in TMJOA acts as an “upstream gatekeeper” driving matrix degradation and inhibiting cartilage synthesis [49], injectable hydrogels can serve as immunomodulatory platforms that suppress local inflammation and reshape the immune milieu as a critical first step toward repair. This approach aims to break the vicious pathological cycle and create a permissive environment for subsequent repair. To this end, researchers have employed functionalized natural polysaccharides like hyaluronic acid (HA) or leveraged the paracrine effects of stem cells. For instance, a biomimetic gelatin/HA hydrogel was shown to downregulate pro-inflammatory cytokines and matrix metalloproteinases (MMPs) while modulating macrophage and T-cell responses in a TMJOA model [50]. Furthermore, intra-articular injection of dental pulp stem cells (DPSCs) as a cell suspension, rather than as a hydrogel formulation, has been reported to delay disease progression by reducing cluster of differentiation 4-positive (CD4+) T-cell infiltration and inhibiting the signal transducer and activator of transcription 1 (STAT1) pathway [51].

However, these strategies face notable limitations. The evidence is often derived from single, chemically induced animal models, whose inflammatory patterns differ from the chronic, mechanically driven nature of human TMJOA. Moreover, most studies focus on short-term improvements in inflammatory markers, while the long-term mechanisms governing the transition from inflammation control to a stable pro-regenerative state remain unclear [48]. Therefore, the true value of these immunomodulatory strategies in TMJOA regeneration may not lie in direct structural reconstruction but rather in their role as “path-clearers”—creating a microenvironment where subsequent pro-anabolic signals can effectively function.

### 5.2. Hydrogels as Antioxidant and Microenvironment-Responsive Systems

Oxidative stress and inflammation create a self-amplifying loop that inflicts sustained damage on cells and the extracellular matrix [52]. Designing “smart” hydrogels that can sense and neutralize local reactive oxygen species (ROS) is therefore aimed at enhancing the spatiotemporal precision of therapy for on-demand intervention. ROS-responsive hydrogels are a primary strategy toward this goal. For example, by incorporating ROS-labile linkages, a hydrogel developed by Kuang et al. [47] underwent triggered degradation in a high-ROS environment, concomitantly releasing fibroblast growth factor 18 (FGF18). This design successfully coupled antioxidant defense with controlled pro-anabolic signaling. Another study integrated catalase-mimicking nanozymes into an HA network, which continuously scavenged ROS and polarized synovial macrophages toward the anti-inflammatory M2 phenotype [38].

Although these works demonstrate the feasibility of responsive therapeutic platforms, their translation remains constrained by several bottlenecks. It is yet to be confirmed whether the ROS-response thresholds validated in vitro align with the complex and dynamic ROS levels within the in vivo TMJ cavity [53] Furthermore, the long-term biosafety and metabolic pathways of novel materials like nanozymes are critical questions that must be answered before clinical consideration. For TMJOA, the profound significance of this responsive design lies in its potential to offer a more efficient intervention for “acute flare-ups,” which are often triggered by fluctuations in joint loading [54], thereby avoiding the side effects associated with continuous, high-dose drug administration.

### 5.3. Hydrogels as Pro-Anabolic and Cell-Laden Scaffolds for Tissue Engineering

Once inflammation and oxidative stress are initially controlled, injectable hydrogels can function as pro-anabolic and cell-laden scaffolds that deliver inductive cues, support seed cells, and activate resident-cell metabolism for structural repair. This is typically achieved by loading hydrogels with small-molecule drugs (e.g., Kartogenin, KGN), growth factors, or regenerative cells. For example, Song et al. [42] utilized KGN-modified GelMA microspheres loaded with DPSCs and observed improved cartilage structure in a TMJ defect model. This demonstrates that co-encapsulating “seed cells” with “chemical cues” in hydrogel microcarriers is a viable approach for promoting in situ cartilage repair.

However, a common limitation of such studies is their reliance on short-term histological improvements as primary endpoints, often without sufficient assessment of the long-term functional stability and mechanical integration of the neo-tissue. More importantly, the TMJ condyle is covered by fibrocartilage rather than hyaline cartilage. Unlike hyaline cartilage, mandibular condylar cartilage contains a more complex extracellular matrix with region-specific distribution of both type I and type II collagens [55]. Accordingly, many current hydrogel-based inductive strategies may be evaluated using canonical chondrogenic markers such as COL2A1, SOX9, and ACAN, which are informative but may not fully capture the requirements for functional fibrocartilage regeneration in the TMJ [56,57,58]. This potential phenotypic mismatch may lead to neo-tissue that is structurally and mechanically suboptimal for the unique loading environment of the TMJ. Therefore, the translational prospects of this strategy depend not merely on achieving structural filling, but on advancing toward functional fibrocartilage reconstruction—a key distinction between TMJ repair and cartilage repair in larger joints.

### 5.4. Hydrogels as Sustained-Release Carriers for Bioactive Factors

As drug and bioactive-factor carriers, injectable hydrogels are particularly valuable for biomacromolecules such as exosomes and growth factors, whose therapeutic efficacy after direct intra-articular injection is often limited by rapid synovial clearance. Incorporating these agents into hydrogel-based sustained-release systems is therefore intended to prolong intra-articular residence and convert short-lived stimulation into more durable biological regulation. Through physical encapsulation or chemical crosslinking, hydrogels can function as local reservoirs for bioactive cargos. For example, encapsulation of DPSC-derived exosomes within an HA-based hydrogel has been reported to improve subchondral bone preservation compared with exosome injection alone [24]. Likewise, bilayer sustained-release systems such as HA/Wnt16@MSN have shown enhanced chondroprotective effects in animal models [59]. Together, these studies support the feasibility of using hydrogel-mediated sustained release to potentiate the effects of regenerative biomolecules.

Despite this promise, several limitations constrain the interpretation and translation of current findings. First, most release profiles are characterized in simplified in vitro systems that do not recapitulate the repetitive functional loading, rapid synovial turnover, and enzymatic degradation present in the TMJ cavity. As a result, the relationship between in vitro release behavior and in vivo pharmacokinetics remains insufficiently defined. Second, follow-up durations in most studies remain relatively short, making it difficult to determine whether the observed benefits reflect transient tissue protection or true long-term functional regeneration. Third, the delivery matrix itself may alter the biodistribution, local retention, and bioactivity of the therapeutic cargo, yet these effects are still poorly understood. Therefore, while sustained-release design represents a key strategy for enhancing the therapeutic value of bioactive factors in TMJOA, its translational significance will depend on establishing TMJ-relevant release validation methods and demonstrating durable biological benefit in long-term in vivo models.

From a translational dental perspective, these regenerative scaffold systems are best understood at present as advanced preclinical strategies for disease modification, rather than as near-term intra-articular solutions ready for routine clinical implementation.

### 5.5. Hydrogel Matrices for 3D Culture and Disease Modeling

Beyond their direct intra-articular use, hydrogels can serve as extracellular-matrix-mimetic matrices for three-dimensional cell culture and disease modeling [60]. Compared with conventional two-dimensional culture, hydrogel-based matrices provide tunable stiffness, viscoelasticity, degradability, and biochemical presentation, thereby supporting a more physiologically relevant microenvironment [61]. In osteoarthritis research, these platforms have mainly been developed for large joints such as the knee, where they are used to model inflammatory stimulation, oxidative stress, mechanical loading, and cell–matrix interactions.

However, TMJOA-specific hydrogel-based three-dimensional culture and organoid-like modeling platforms remain at an early stage, and relevant studies are scarce [48]. Findings from large-joint osteoarthritis models cannot be directly extrapolated to the TMJ because mandibular condylar cartilage is fibrocartilaginous and is exposed to a distinct rotational–translational loading environment. Therefore, although these systems are not yet mature TMJOA-specific models, they may serve as useful preclinical tools for screening hydrogel formulations, evaluating drug release and cytocompatibility, and investigating TMJ-relevant disease mechanisms.

## 6. Hydrogels as Joint Lubricants and Mechanical Buffers

Beyond serving as active, multifunctional platforms for biological integration, the design rationale for injectable hydrogels in TMJOA has also evolved from simple viscosupplementation toward a multi-dimensional synergy of lubrication, mechanical buffering, prolonged residence, and sustained delivery. The core principle of this approach is to leverage the intrinsic physical properties of the hydrogel to reduce friction and absorb shock during the frequent repetitive movements of the joint. The role of these materials is thus expanding from purely symptomatic relief to direct intervention in the disease process. Compared to regenerative scaffolds that require bioactive payloads, materials designed for physical functions present a more straightforward path to clinical translation and are more amenable to scalable manufacturing and quality control, granting them greater near-term clinical feasibility [62,63].

The starting point for this evolution remains the clinical use of hyaluronic acid (HA) as a viscosupplementation strategy. In TMJOA, however, the clinical efficacy of intra-articular HA remains controversial. Although HA has been used to improve lubrication and viscoelastic properties within the joint cavity, recent evidence syntheses have not consistently demonstrated superiority over placebo injection in terms of pain relief and functional improvement [33,64,65]. Earlier systematic reviews suggested potential benefits for pain and mandibular mobility, but the overall evidence remains heterogeneous across study designs, comparators, and outcome measures. Therefore, in the context of this review, HA should be regarded not simply as an established effective therapy, but as an important conceptual and biomaterial precursor for subsequent injectable hydrogel systems designed to enhance intra-articular retention, tribological performance, and local biomechanical support [34]. Beyond the debated clinical efficacy of conventional HA injection in TMJOA, the mechanistic rationale for lubrication-oriented hydrogel design is still derived largely from studies in larger joints such as the knee (i.e., generalized OA). For instance, in knee OA models, biomimetic-coated microspheres have been shown to reduce the friction coefficient by nearly 30% [45], while a brush-like composite nanofiber demonstrated chondroprotection and inflammation suppression for up to 8 weeks in a rat model [29]. These findings support the general concept that friction reduction may contribute to cartilage protection, but they do not establish that the same degree of tribological benefit can be directly reproduced in the temporomandibular joint, where the articular surface is fibrocartilaginous, the cavity is small, and the loading pattern is dominated by complex rotational–translational motion [66].

Direct evidence for TMJ-specific lubrication strategies remains limited. While one report showed that self-assembling microparticles exhibited a synergistic lubricating and anti-inflammatory effect in a TMJOA model [30], the follow-up was only 4 weeks, and the high injection frequency (weekly) failed to substantiate long-term material residence or durability of the therapeutic effect. Furthermore, even when novel dual-action materials like ketoprofen-loaded hyaluronic acid hydrogels demonstrate promising rheological feasibility, their evaluations are often severely restricted to short-term (24-h) in vitro cellular assays without any complex in vivo biomechanical validation [34]. Thus, the current literature supports the feasibility of lubrication-oriented intervention in TMJOA, but not yet its long-term functional superiority under clinically relevant conditions [29,40].

Unlike lubrication, the mechanical buffering function relies more on a material’s appropriate viscoelastic profile than on sheer stiffness. An ideal hydrogel should exhibit shear-thinning properties for ease of injection—typically maintaining a low precursor viscosity (e.g., ~2.3 × 10^3^ cP) that rapidly increases upon gelation at body temperature to form a stable depot (exceeding 6.9 × 10^4^ cP) [36]—rapid structural recovery post-injection for stable gel formation, and the ability to absorb impact and attenuate stress. Under physiological loading, this buffering is mediated by an appropriate storage modulus (G′) and energy dissipation capacity, which is characterized by the loss modulus (G″) or the loss tangent (tan δ = G″/G′) [67]. Depending on the crosslinking chemistry, the half-life of stress relaxation (τ_1/2_) of these joint-targeted networks can be finely tuned across orders of magnitude, ranging from fast-relaxing regimes (τ_1/2_ < 10 s for imine or boronate bonds) to highly elastic, slow-relaxing regimes (τ_1/2_ > 1000 s for hydrazine or oxime chemistries) [61] to match the dynamic loading frequencies of articular joints. However, in current TMJOA research, the evidence base for mechanical buffering remains substantially weaker than that for lubrication. In most cases, buffering capacity is inferred from rheological or viscoelastic measurements rather than directly linked to long-term in vivo functional benefit [68].

Within the actual joint cavity, materials face the combined challenges of synovial dilution, extravasation, and premature degradation. To address this, researchers have explored various engineering strategies, such as in situ crosslinking or microparticle-based systems to enhance residence time [43], and the incorporation of dynamic covalent bonds (e.g., boronate esters) to balance injectability with mechanical durability [30]. Furthermore, achieving long-term sustained release of complex therapeutic agents from the crosslinked network provides a viable path to enhance long-term efficacy across all four dimensions: lubrication, buffering, residence, and delivery [40]. Nevertheless, whether these material-level improvements can be translated into measurable buffering benefit in the TMJ remains insufficiently validated [36].

In summary, utilizing hydrogels as lubricants and mechanical buffers represents a pathway for TMJOA treatment that is centered on physical intervention and is closer to clinical translation. The current body of research reveals a clear asymmetry of evidence: improvements in lubrication are supported by preliminary in vivo studies, although their long-term relevance remains uncertain, whereas the biological significance of mechanical buffering is still largely a reasonable inference based on in vitro rheological performance. Accordingly, current studies remain limited by the lack of TMJ-relevant evaluation frameworks that directly connect tribological and viscoelastic parameters with clinically meaningful outcomes, such as maximal mouth opening, mandibular kinematics, chewing-related function, and pain-related behavioral improvement [69,70].

Accordingly, among current hydrogel-based approaches, lubrication- and buffering-oriented acellular systems may be the most compatible with existing conservative TMJOA management workflows and therefore the most plausible candidates for near-term clinical translation [41].

## 7. Clinical Translation and Commercialization

As outlined in the Introduction, the short duration of action of conventional intra-articular agents necessitates repeated injections, and the central translational value of durable hydrogel-based delivery systems lies in reducing injection frequency and prolonging local therapeutic action [7,35]. The supporting evidence, however, remains overwhelmingly preclinical: in the only systematic review of drug-delivery systems tested in TMJOA models, just one of the included studies was clinical while the remainder were performed in animals, predominantly rats [35]. More broadly, most hydrogel studies for TMJ disease have been conducted in vitro or in animal models, leaving clinical feasibility uncertain [7], and the clinical treatment of TMJOA is still described as being in its infancy [6].

The translational outlook diverges markedly between the two principal categories of hydrogel. Regenerative, cell- and biologic-based systems, such as stem-cell-laden or exosome-loaded hydrogels [28,42], face additional barriers: they depend on cell sources of limited availability [7], and the integration of seed cells, bioactive factors, and scaffold materials remains unresolved, with no definitive regenerative cure achieved to date [11]. For drug-loaded systems, controlling burst release and maintaining the stability and bioactivity of the cargo within the hydrogel matrix are further recognized challenges [7]. By contrast, acellular functional hydrogels that extend the established viscosupplementation approach align more closely with the intra-articular treatment workflow already used clinically and avoid these cell-sourcing and integration hurdles.

## 8. Conclusions and Future Outlook

Injectable hydrogels for TMJOA are currently advancing along two translational pathways: regenerative scaffold systems aimed at structural restoration and functional biomaterial systems designed to improve lubrication, buffering, and local disease control. Although regenerative approaches hold substantial theoretical promise for definitive repair, their progress toward clinical application remains constrained by the manufacturing, safety, and regulatory barriers detailed in Section 7. By contrast, acellular multifunctional hydrogels that combine viscosupplementation with anti-inflammatory or antioxidative activity appear more compatible with current conservative TMJOA treatment workflows and may therefore represent the most realistic near-term translational strategy.

For these materials to move from preclinical promise to clinical relevance, future studies must extend beyond short-term efficacy and address long-term biosafety, intra-articular durability, degradation-product fate, repeat-dose feasibility, and manufacturing reproducibility under TMJ-relevant conditions. Particular attention should be given to whether incomplete clearance of degradation products or retained polymer fragments may contribute to persistent synovial inflammation or foreign-body-type responses, depending on the material chemistry and degradation profile [41].

From a dental clinical perspective, the future role of injectable hydrogels is unlikely to be as standalone biomaterials, but rather as components of an integrated management strategy that links symptom control, intra-articular microenvironment stabilization, and, where appropriate, staged structural repair. In this context, functional acellular hydrogels may be most valuable in the earlier phases of conservative management, whereas regenerative platforms may be reserved for later-stage biological augmentation once safety, durability, and functional benefit have been more convincingly established. Overall, the clinical value of injectable hydrogels for TMJOA will depend not only on advances in biomaterial design, but also on their ability to deliver measurable functional benefits within realistic dental and oral medicine practice settings.

## Figures and Tables

**Table 1:** Overview of representative injectable hydrogel systems for TMJOA.

Polymer Type	Components	Cargo	Effects	Mechanism(s)	Model	Advantages	Ref.
Natural (HA)	HA, VS	DPSC-derived exosomes	Attenuates damage-mediated subchondral bone loss	Anti-inflammatory	Rat TMJOA	Stable local exosome concentration; single injection	[28]
Natural (HA)	HA-HYD, HA-ALD; ε-polylysine-coated catalase-mimic nanozyme	—	Eliminates excess ROS; upregulates chondrogenesis; reduces pro-inflammatory cytokines; protects condylar cartilage and subchondral bone	ROS scavenging; M2 polarization	Rat TMJOA	Stable nanozyme; long-lasting ROS clearance; high biocompatibility	[38]
Natural (HA)	HA–GA, TSPBA	FGF18	Reduces inflammation; facilitates cartilage repair	ROS scavenging; FGF18 and gallic acid delivery	Rat TMJOA	ROS-responsive; dual therapeutic functions	[39]
Natural (HA)	HP (HA-based microparticles)	Luteolin	Protects chondrocytes; alleviates subchondral bone lesions; suppresses inflammation; promotes matrix regeneration	Antibacterial; lubricating; antioxidant; anti-inflammatory	Rat UAC TMJOA	Good lubrication; high drug loading; antioxidant	[30]
Natural (HA)	HA	BMSC-conditioned medium	Improves disc, fibrocartilage and trabecular bone; elevates collagen; inhibits NF-κB	Anti-inflammation	Rat TMJOA	Innovative formulation; well tolerated; safe	[40]
Natural (gelatin/HA)	TGI/HA (tilapia gelatin I/HA)	—	Reduces ECM-degrading enzymes and inflammatory factors; induces M2 polarization and Th2 differentiation	Immunomodulation; cartilage regeneration	Rat TMJOA	Multifunctional; low-cost; easy administration; clinically translatable	[1]
Natural (gelatin/HA/CS)	TGI/HA-CS	—	Inhibits macrophage inflammasomes; reduces ECM degradation; boosts disc-cell proliferation and SMSC fibrochondrogenesis; repairs large disc defects	Immune-microenvironment regulation; in situ disc regeneration	Rat TMJOA; minipig perforated disc	High-strength; multifunctional; light-curing; double-network; large-animal validated	[41]
Natural (GelMA)	Fish-skin gelatin methacryloyl microspheres	DPSCs, KGN	Promotes DPSC chondrogenic differentiation; repairs cartilage defect	Sustained chondrogenic stimulation (KGN microspheres)	Rat TMJOA	Strong chondrogenic induction; tunable differentiation	[42]
Natural (GelMA)	GelMA (superwetting microspheres)	rBMSCs + TGF-β	Accelerates cell spreading; sustained factor release; reconstructs irregular cartilage defects	Superwetting; sustained TGF-β delivery	Rat TMJOA	Superwetting; sustained delivery	[43]
Natural (GelMA)	GelMA	DPSCs	High healing scores; eliminates inflammation; promotes subchondral bone regeneration (no cartilage regeneration)	Facilitates bone regeneration	Rabbit TMJ condylar defect	Complete in vivo degradation; no inflammatory response	[44]
Composite (GelMA/MPC)	GelMA, DMA-MPC	DS (diclofenac sodium)	Enhances lubrication; upregulates cartilage anabolism and downregulates catabolism	Anti-inflammatory; antioxidant	Rat OA	Excellent lubrication; controllable sustained release	[45]
Composite (alginate/PEI)	OSAP, PPCA	BGN@Be	Inhibits angiogenesis and neurogenesis; alleviates OA pain	Scavenges exRNA; inhibits neurovascularization	Mouse UAC TMJOA	Self-healing; pH/ROS dual-responsive; on-demand delivery	[25]
Synthetic (ROS-responsive)	RDGel	DPSCs	Upregulates cartilage matrix; remodels subchondral bone; alleviates oxidative stress; induces M2 polarization	Anti-apoptosis; immunomodulation; anti-inflammation; antioxidation	Rat TMJOA	ROS-responsive matrix; stem-cell regeneration; good biocompatibility	[46]
Synthetic (PLCL-PEG-PLCL)	PLCL-PEG-PLCL	Nell-1	Promotes chondrogenic expression; suppresses inflammation; restores subchondral bone and joint surface	Nfatc1–Runx3 signaling	Collagenase-induced TMJOA (rabbit)	Wide gelation temperature range; high hydrolytic stability	[32]
Synthetic (polymeric nanoparticle)	TePNs	TCA (triamcinolone acetonide)	Blocks OA progression; restores damaged cartilage	Anti-inflammation	Rat knee OA	Easy preparation; high drug loading; ~1-month sustained release	[44]

## Data Availability

Data sharing is not applicable to this article as no datasets were generated or analyzed during the current study.

## Abbreviations

3D	three-dimensional
ACAN	aggrecan
ADAMTS	a disintegrin and metalloproteinase with thrombospondin motifs
CD4+	cluster of differentiation 4
COL2A1	collagen type II alpha 1 chain (type II collagen)
DMOADs	disease-modifying osteoarthritis drugs
DPSCs	dental pulp stem cells
ECM	extracellular matrix
F127 (F127DA)	Pluronic F127/poloxamer 407 (F127DA, F127 diacrylate)
FGF18	fibroblast growth factor 18
GelMA	gelatin methacryloyl
G′	storage modulus
G″	loss modulus
HA	hyaluronic acid
KGN	kartogenin
MMPs	matrix metalloproteinases
MSN	mesoporous silica nanoparticle
Nell-1	NEL-like protein 1
NSAIDs	nonsteroidal anti-inflammatory drugs
OA	osteoarthritis
PEG	poly(ethylene glycol)
PLCL	poly(L-lactide-co-ε-caprolactone)
PLGA	poly(lactic-co-glycolic acid)
ROS	reactive oxygen species
SilMA	methacrylated silk fibroin
SOX9	SRY-box transcription factor 9
STAT1	signal transducer and activator of transcription 1
tanδ	loss tangent (damping factor)
TMD	temporomandibular disorders
TMJ	temporomandibular joint
TMJOA	temporomandibular joint osteoarthritis
Wnt16	Wnt family member 16
